# Designing of PLA scaffolds for bone tissue replacement fabricated by ordinary commercial 3D printer

**DOI:** 10.1186/s13036-017-0074-3

**Published:** 2017-10-16

**Authors:** Aleš Gregor, Eva Filová, Martin Novák, Jakub Kronek, Hynek Chlup, Matěj Buzgo, Veronika Blahnová, Věra Lukášová, Martin Bartoš, Alois Nečas, Jan Hošek

**Affiliations:** 1 Department of Instrumentation and Control Engineering, Faculty of Mechanical Engineering, Czech Technical University in Prague, Technická 4, 166 07 Prague 6, Czechia; 2 Institute of Experimental Medicine of the Czech Academy of Sciences, Vídeňská 1083, 14220 Prague 4, Czechia; 3 Second Faculty of Medicine, Charles University, V Úvalu 84, 150 06 Prague 6, Czechia; 4 Department of Mechanics, Biomechanics and Mechatronics, Faculty of Mechanical Engineering, Czech Technical University in Prague, Technická 4, 166 07 Prague 6, Czechia; 5 University Centre for Energy Efficient Buildings, Třinecká 1024, 273 43 Buštěhrad, Czechia; 6 Faculty of Science, Charles University, Albertov 6, 12843 Prague 2, Czechia; 7 Department of Stomatology, First Faculty of Medicine, Charles University and General University Hospital in Prague, Kateřinská 32, 12801 Prague 2, Czechia; 8 University of Veterinary and Pharmaceutical Sciencies Brno, Palackého tř. 1946/1, 612 42 Brno, Czechia

**Keywords:** Tissue engineering, Scaffold, Bio-fabrication, 3D printing, Rapid prototyping, Polylactic acid, Fused deposition modelling, Rebel II

## Abstract

**Background:**

The primary objective of Tissue engineering is a regeneration or replacement of tissues or organs damaged by disease, injury, or congenital anomalies. At present, Tissue engineering repairs damaged tissues and organs with artificial supporting structures called scaffolds. These are used for attachment and subsequent growth of appropriate cells. During the cell growth gradual biodegradation of the scaffold occurs and the final product is a new tissue with the desired shape and properties.

In recent years, research workplaces are focused on developing scaffold by bio-fabrication techniques to achieve fast, precise and cheap automatic manufacturing of these structures. Most promising techniques seem to be Rapid prototyping due to its high level of precision and controlling. However, this technique is still to solve various issues before it is easily used for scaffold fabrication.

In this article we tested printing of clinically applicable scaffolds with use of commercially available devices and materials. Research presented in this article is in general focused on “scaffolding” on a field of bone tissue replacement.

**Results:**

Commercially available 3D printer and Polylactic acid were used to create originally designed and possibly suitable scaffold structures for bone tissue engineering. We tested printing of scaffolds with different geometrical structures. Based on the osteosarcoma cells proliferation experiment and mechanical testing of designed scaffold samples, it will be stated that it is likely not necessary to keep the recommended porosity of the scaffold for bone tissue replacement at about 90%, and it will also be clarified why this fact eliminates mechanical properties issue. Moreover, it is demonstrated that the size of an individual pore could be double the size of the recommended range between 0.2–0.35 mm without affecting the cell proliferation.

**Conclusion:**

Rapid prototyping technique based on Fused deposition modelling was used for the fabrication of designed scaffold structures. All the experiments were performed in order to show how to possibly solve certain limitations and issues that are currently reported by research workplaces on the field of scaffold bio-fabrication. These results should provide new valuable knowledge for further research.

## Background

To repair damaged tissues and organs, tissue engineering currently utilizes artificial supporting structures called “scaffolds”, which serve as carriers of cell cultures and control their growth. Scaffolds are fabricated as porous structures of pre-defined shapes. Their structure properties include external geometry, porosity, porous interconnectivity, individual pore size, and surface area [1]. Scaffolds are used in particular as carriers for growing bone tissue, cartilage, ligaments, skin, blood vessels, nerves and muscles [2]. They are also used as carriers for the controlled delivery of drugs and proteins. Scaffolds are prepared using biodegradable materials, allowing the material gradually disintegrates (degrades) after the formation of a new tissue or organ. Scaffolds are seeded with suitable cells (depending on the type of tissue) in vitro and then implemented in vivo into the place of damage. There, through the porous structure of the scaffold a cell proliferation occurs, which enables the formation of a new tissue. Materials currently used for scaffold manufacturing are split into several types; entirely synthetic materials, natural materials, ceramics, and their combinations. Natural fibres used in scaffolding include collagen, the protein that creates the majority of extracellular matrix; alginate, a plant polymer derived from algae; chitosan, derived from chitin found in insects and fibrin gel [3]. Synthetic materials allow for a better control of chemical, physical and mechanical properties, as well as degradation rate. In addition, fabrication methods can process synthetic materials into scaffolds of desired porosity, morphologies, and anisotropies with improved cell attachment and migration. The disadvantages of synthetic scaffolds are possible toxicity and undesired inflammatory responses. The synthetic materials that scaffolds are usually made of are polymeric. The most popular polymers are linear aliphatic polyesters. This group includes polyglycolic acid (PGA), polylactic acid (PLA), and their co-polymers polylactic co-glycolic acid (PLGA). The degradation of PLA, PGA and PLA/PGA copolymers generally involves random hydrolysis of their ester bonds. PLA degrades to form lactic acid which is normally present in the body [4]. Scaffolds can be also created by combining synthetic and natural materials [5]. Ceramic materials are usually used in combination with polymers to substitute tissue with an expectancy of high resilience [6]. In recent years, technological development of scaffolds uses several approaches so-called bio-fabrication. However, many of those fabrication techniques have not yet achieved adequate results to be applied in current clinical practice. Most of the techniques currently used for scaffold fabrication provide low quality as for the pores sizes and their interconnectivity within the scaffold structure. One of the most promising techniques for an “ideal” scaffold structure fabrication is Rapid prototyping due to its excellent control over the geometry of the created sample [7]. While industrial 3D printers have reached extremely high resolution in the past few years, the advancements in machine capability have not transferred to the use with biomaterials. These systems unfortunately are not optimized for biomaterials of interest for in vitro and in vivo studies [8]. Clinical application is limited due to high machine cost, design and fabrication time involved. High processing temperatures in certain techniques limit their ability to process temperature-sensitive polymers with bioactive component. Another limitation of a high temperature is possibility to affects the mechanical strength [9]. One of the most promising ways of automated bio-fabrication appears especially in the principle of the Fused Deposition Modelling (FDM) [10], which is mainly used in cases of synthetic polymers applications.

Regular inner and outer structure of the scaffold is another important property. Sufficient and regular porosity is required for uniform cell proliferation both in the space of scaffolds and in time. The speed of cell proliferation and degradation of the material should ideally be uniform. Current studies report that ideal scaffold porosity should be around or more than 90% (especially for bone tissue engineering) and pores should provide good interconnectivity to ensure good proliferation of cells [11]. Unfortunately, porosity reduces mechanical properties such as compressive strength, and increases the complexity for reproducible scaffold manufacturing. Mechanical properties constitute another important feature of the scaffold. This importance has multiple reasons; growing cells may exert force, and certain cell types such as fibroblasts generate substantial force, a mechanically weak scaffold might be broken down under the load of these forces and change the shape of the final tissue structure [12].

Important for growing tissue is the control of the proliferation and the nutrient transfer characteristics within the scaffold structure [13]. One of the future challenges in bone tissue engineering is to design and to manufacture biodegradable scaffolds with a homogeneous growth rate over their entire volume, using pore size gradients or specific distributions of embedded growth factors. This requires manufacturing processes with higher resolution and bio-fabrication capabilities [14]. Öchsner et al. suggested in their review how to overcome current limitations and move the current scaffold fabrication by Rapid prototyping to the next frontier. First step is the continuous improvement of Rapid prototyping machines to produce mass production with cost effective precise scaffolds through enhancing machines resolution, accuracy, trapped liquid or loose powder removal techniques and developing methods for direct placements of bioactive components such as cells and proteins within the 3D structures. Finally, further improvements in a scaffold’s internal and external architecture in addition to the incorporation of material heterogeneity within the scaffold structure are needed to obtain the optimal scaffold design [15]. Based on current issues described above it may be stated that the topic is very much in the focus and appears to be frequently investigated by research workplaces that are focused on scaffolding in tissue engineering.

## Scope of the research

This research deals with the hypothesis, whether it would be possible to overcome the aforementioned technical limitations and fabricate, or rather print functional and clinically applicable scaffolds using current, cheap and commercially available devices and materials. Experiments described in this article are focused on fabrication of scaffolds that might be eventually used on field of bone tissue replacement. The basic premise was the use of ordinary and commercially available 3D printer and cheap pure PLA material, which is usually used as a filament for such 3D printers. PLA is a bio-degradable material and is normally used in tissue engineering for bone tissue replacement purposes. Current, commercially available and cheap (300–1000€) 3D printers could reach good quality resolution of printing around 0.3 mm. This could provide the possibility to use them at least for bone tissue engineering, where the recommended pore size of the scaffold is 0.2–0.35 mm [16]. Such a 3D printer could produce precise layer by layer structures that provide good and regular interconnectivity between pores and also have good mechanical properties. Another advantage of these printers is that there are biodegradable materials as a printing “feed” already in use and their price is low. One of them is PLA. The reported foam scaffolds with proper cell ingrowth and nutrition diffusion had porosity around 90% [11]. We would like to test 3D printed scaffolds with lower porosity and structure for their potential in tissue engineering. Moreover, we want to test the impact of different porosity on the mechanical properties of the scaffolds as we logically expect the worse mechanical properties in case of the higher porosity level. Young′s modulus of printed scaffolds will be determined and compared with scaffolds made from the same material by different or by similar approaches for the same purpose, the bone tissue replacement. In order to confirm/reject proposed hypotheses and to obtain adequate results, two types of scaffold structure were designed and printed, osteosarcoma cells proliferation through both scaffold structures were investigated and basic mechanical tests were performed.

There exist previous studies employing 3D printer for scaffold design [17–19]. Our research novelty is focused on assessment of newly designed scaffold structures that have not yet been used. We reached successful results of equal proliferation and osteoconduction in the scaffold with only 30% porosity compared to scaffold with 50% porosity (recommended porosity is 90% [11]). This may eliminates mechanical properties issues reported in case of scaffolds with high porosity. We also proved successful cell proliferation and osteoconduction in the scaffold type with two time larger pores than recommended for bone tissue engineering scaffolds [16].

## Methods

### Scaffold structures

**Fig. 1:**
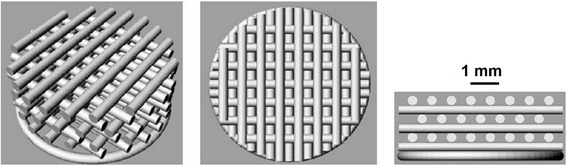
Scaffold structure ST1. The porosity of ST1 scaffold was expected around 30% and intended diameter of the fibre is 0.35 mm and pore size 0.35 mm

**Fig. 2:**
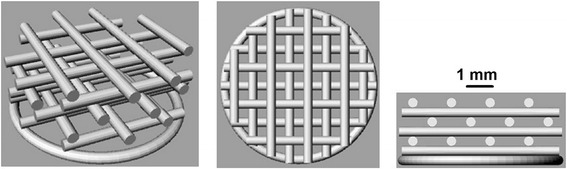
Scaffold structure ST2. The porosity of ST2 scaffold was expected around 50% and intended diameter of the fibre is 0.35 mm and pore size 0.7 mm

Important parameters which scaffold should meet for a proper cell proliferation is sufficient and regular porosity, and imitation of the original architecture of tissue or organ that needs to be regenerated. According to these conditions 2 types of scaffold structures for bone tissue regeneration were designed and printed. The reasons of different inner structures of both scaffolds are as follows:

- **Scaffold ST1** – Presumption that the scaffold will be seeded by cells from the top. Therefore individual fibres need to overlap each other vertically in each second layer to prevent the cells “fall” down through the scaffold structure (see - scaffold in Fig. 1).

- **Scaffold ST2** – Porosity is cca 50–60% higher then in case of ST1 in order to determine whether the cells attach individual fibres even if there are vertical gaps between layers (see - scaffold in Fig. 2).

### 3D printing method

Freeware Repetier Host (http://www.repetier.com/download/) was used for generation of G-code. The printing process is not designed for such a small objects such as the scaffolds. The generated G-code was therefore not entirely correct and was not usable directly for printing. It had to be manually modified. Only the first two layers of the generated G-code were taken for scaffold ST1 and the ﻿first three layers for scaffold ST2. The code was cleaned by removing any unwanted movements so that one fiber is printed without any interruption. The printing speed was hand optimized to a feedrate of 1080 mm/min for both ST1 and ST2. The non-printing moves were set to 7800 mm/min. The layer height was set to 0.2 mm. The first two or three layers respectively were then recopied to a different height until the desired scaffold height was reached. The filament flow rate was also manually adjusted to 130% of the nominal value. Finally the code for one scaffold was multiplied to print multiple scaffolds at a time. The printing time for a batch of 4 scaffolds was about 15 min.

#### Basic technical parameters of the device (according to the manufacturer)

Printing space: 190x190x180 mm; Filament Diameter: 1.75 mm; Inner nozzle diameter: 0.2 mm; Accuracy: X and Y resolution (theoretical) 6.25 μm. Z axis resolution (theoretical) 0.156 μm.

### Scaffolds structure measurement

We checked printed scaffold porosity with two independent methods – based on known density of used PLA (1.25 g/cm^3^) and its volume and using X-ray microtomography. At the first we calculated the theoretical weight of each particular scaffold without any pores. The real weight of each scaffold was then proportionaly compared to the calculated weight (without pores) and thus the porosity was determined. Furthermore, three ST1 samples (ST1a, ST1b and ST1c) and three ST2 samples (ST2a, ST2b and ST2c) were scanned using X-ray microtomography (Bruker SkyScan 1272, max. Resolution 0.5 μm). The scanning was performed to confirm the method mentioned above and exclude the presence of closed pores (air bubbles). Both standard porosity (%) and closed porosity (%) were evaluated as ratio of volume of all or closed pores and total volume. Another evaluated parameters were: number of closed pores (1), surface of the samples (mm^2^), surface to volume ratio (mm^−1^), average thickness of the fibres (mm) and distribution of the thickness in graph (mm to % of volume). All the results are available in results chapter of this article.

### Scanning electron microscopy (SEM)

PLA samples were glued on aliminium stubs and sputter-coated with a platinum layer using a Quorum Q150R (Quaorum Technologies Ltds). The samples were examined in a Vega 3 SBU (Tescan) scanning electron microscope in the secondary electron mode at 30 kV. The mean fiber diameter was calculated by image analysis in the ImageJ program. A figure of scanned scaffold is presented in the results chapter.

### PLA properties measurement

Verification of processed PLA material properties were performed with FTIR-IR analyzer, Surface zeta potential measurement, Contact angle measurement and Molecular weight and polydispersity measurement. Results are presented in the results chapter of the article.

### FTIR-IR spectrum measurement

Chemical identity of the material was analysed using FTIR (IRAffinity 1, Shimazu). Attenuated total reflactance (ATR) method was used for analysis of PLA 3D printed samples. The 3D printed scaffold was melted at 200 °C to produce film on glass coverslip. The spectrum of thin film was measured in range from 800 to 4000 cm-1 as 20 independent measurements. The Happ Gazel appodization was used for spectrum deconvolution.

### Surface zeta potential measurement

Zeta potential was measured on Zetasizer ZS (Malvern Instruments Ltd., UK) using surface-zeta potential cell. Standard silicon particles with zeta potential of −42.2 mV were used as a tracer material. The PLA sample was attached to the sample holder. The zeta potential was measured using standard protocol. The sample was measured in 3 measurements with 15 runs in each measurement. Temperature was set to 25 °C. The surface zeta potential was calculated as change of particle zeta potential as a function of displacement from the surface. The surface zeta potential was calculated in 4 points with displacement of 250 μm. Surface zeta potential was measured from pure PLA plate or PLA plate incubated with 1 mg/mL type I collagen (PLA Col) or with 1 mg/mL hydroxyapatite suspension for 20 min at room temperature (RT).

### Contact angle measurement

Contact angle was evaluated using computer-based instrument SEE Systems (Advex Instruments, Czech Republic). From the distilled water droplet formed on a flat PLA polymer was scanned using a camera, and the contact angle was calculated from 7 independent measurements.

### Molecular weight and polydispersity measurement

Number-average molecular weight (M n) and polydispersity index (M w/M n) of the PLA was determined using gel permeation chromathography with multi-angle light scattering method (GPC-MALS). An instrumental setup included Agilent HPLC 1100 Series instrument with degasser, pump, autosampler, set of two PLgel 5 μm Mixed-C 300 × 7.5 mm columns (Agilent, USA) thermostated to 25 °C and UV-VIS diode array detector in connection with a DAWN HELEOS II multi-angle laser light scattering detector, ViscoStar-II differential viscometer and Optilab T-rEX refractive index detectors (Wyatt Technology, Germany). Both MALS and RI detectors operated at 658 nm. Tetrahydrofurane was used as the mobile phase at a flow rate of 1 mL/min. Sample in THF (concentration 1 mg/mL) was filtered with 0.22 μm filter and injected in the volume of 100 μL. Astra 6.1 software was used for data collection and analysis and Agilent software was used to control the HPLC. The specific refractive increment dn/dc equal to 0.049 for PLA was used for data processing. The specific refractive index values of polylactide were confirmed by a 100% mass recovery.

### Experiments with osteosarcoma cells

Typical methods/assays were used to determine whether osteosarcoma cells are applicable and survive on our scaffold and whether they provide with osteoconduction.

### PLA cytotoxicity test

Before the seeding of MG-63 cells into the scaffolds, the cytotoxicity test of PLA material was performed.

Dense PLA scaffolds were incubated in a Dulbecco’s Modified Eagle’s medium (DMEM, GIBCO) medium supplemented with 10% fetal bovine serum (FBS), 100 IU/ml penicillin and 100 μg/ml streptomycine for 4 days in the same PLA/medium ratio as the standard cell culture (conditioned medium). The conditioned medium was used for the cell cytotoxicity test. 3 T3 fibroblasts were seeded on tissue culture polystyrene (TCP) at the density of 2.5 × 10^3^ cells/well in both the conditioned medium and in the standard culture medium, and cultured for 1, 3, and 5 days in the 96-well plates. The metabolic activity was tested using an MTS test. For the test, 20 μl MTS solution was added into 100 μl medium for 2 h, and the absorbance of 100 μL solution was measured at 490 nm (reference wavelength was 690 nm).

### Cell seeding

Osteosarcoma cell line MG-63 was seeded on both PLA scaffolds at the density 20 × 10^3^ cells and cultured in DMEM medium supplemented with penicillin, streptomycin (100 IU/ml and 100 μg/ml, respectively), L-glutamin and 10% fetal bovine serum in a CO_2_ incubator with 5% CO_2_ at 37 °C for 21 days. Medium was changed every 3–4 days. 4–5 scaffolds were seeded for cell metabolic activity/DNA assay; cells seeded on tissue culture polystyrene (TCP) were used as a control. 3 scaffolds were used for DiOC6(3)/propidium iodide staining and 4 scaffolds for osteocalcin staining.

### Metabolic activity assay

The MTS assay reflects metabolic activity of the cells as well as the cytotoxicity of the scaffolds and is an approved method for cytotoxicity evaluation (ISO 10993–5:2009). Cell metabolic activity is measured by converting MTS by mitochondrial dehydrogenases.

On days 1, 3, 7 and 14 the cell metabolic activity was evaluated using the MTS assay (CellTiter 96® AQueous One Solution Cell Proliferation Assay; Promega). 20 μl MTS solution was added to 100 μl medium with a scaffold and incubated at 37 °C for 2 h. 100 μl solution was transferred into new 96-well plate and the absorbance was measured at 490 nm (reference wavelength was 690 nm).

### Cell proliferation assay

Cell proliferation was evaluated using Quant-iT™ dsDNA Assay Kit (Life Technologies). This method is very sensitive and is able to detect ds DNA amount in a range of 0.2–100 ng per sample and was used in previous experiments (Samples were put into lysate buffer (0.2% *v*/v Triton X-100, 10 mM Tris (pH 7.0) and 1 mM EDTA) and were frozen at −80 °C 1, 3, 7, 14, and 21 days after seeding. After collecting all the samples in 1000 ul lysate buffer, three cycles of thawing, vortexing and freezing at −80 °C were applied. After the third cycle was finished, all samples were immediately measured at ﻿room temperature (RT). The DNA standards were included in the kit. All tested samples were processed at the same time therefore no differences in DNA isolation are expected [20]. DNA was measured according producer instructions at RT. Briefly, 200 μl of Quant-1 T™ dsDNA HS reagent, which was diluted with enclosed buffer, was loaded in a 96-well plate. 10 μl DNA standards were added in doublets into wells. Similarly, 10 μl samples, 4 per group each day were added in doublets into other wells with the reagent and gently mixed. The amount of DNA was evaluated from fluorescence measurement using Multi-Detection Microplate Reader (Synergy HT, λex = 485 nm, λem = 525 nm) and calculated from the calibration curve. DNA was measured using 10 ul sample solution, which is in the range of assay sensitivity, and then calculated to obtain total DNA amount in the samples (total volume was 1000 ul), which was shown in a graph.

### Cell visualization on the scaffolds

Cells on the scaffolds were fixed by frozen methanol (−20 °C) on days 1, 7, 14 after seeding. The scaffolds were twice washed with phosphate-buffered saline, and cell membranes were stained with 1 μg/mL of 3,3′-dihexyloxacarbocyanine iodide (DiOC6(3) (Cat. No. D273, Invitrogen) for 45 min and subsequently, cell nuclei were stained with propidium iodide. The cells were visualized under a confocal microscope (Zeiss LSM 5 DUO) at λexc = 488 nm, λem = 505–550 nm for DiOC6(3) and λexc = 560 nm, λem >575 nm for propidium iodide.

Live/dead staining was performed by staining of viable cells by BCECF-AM and propidium iodide. Viable cells were able to retain BCECF-AM in their cytoplasm. On contrary, dead cells were visualized by incorporation of propidium iodide to free DNA from dead cells. The scaffolds were stained by 2′, 7′- Bis (2-carboxyethyl)-5(6)-carboxyfluoresceinacetoxymethyl ester (BCECF-AM, Sigma Aldrich) diluted 1:100 in medium) was added and incubated for 35 min at 37 °C and 5% CO2 for live cells detection. It was then rinsed with PBS (pH 7.4); propidium iodide (5 μg/ml in PBS pH 7.4) was added for 6 min, rinsed with PBS (pH 7.4) The cells were visualized under a confocal microscope (Zeiss LSM 5 DUO) at λexc = 488 nm, λem = 505–550 nm for BCECF-AM and λexc = 560 nm, λem >570 nm for propidium iodide.

PLA samples seeded with MG-63 on day 2 were washed in PBS and fixed in 2.5% glutaraldehyde for 2 h at 4 °C. The samples were after that dehydrated in ethanol ranging from 35%–100%. Hexamethyldisilazane (Sigma-Aldrich) was added to dry the scaffolds. Scaffolds were analyzed using Vega 3 Tescan as described in chapter “Scaffolds structure measurement”.

### Production of osteogenic markers

Evaluation of osteogenic marker production was based on the visualization of type I collagen and osteocalcin which are markers of osteogenic differentiation. Immunohistochemical staining was performed using mouse monoclonal antibody against osteocalcin (overnight, 2–8 °C, dilution 1:200, ab13420, Abcam) or mouse monoclonal antibody against type I collagen (concentrate, overnight, 2–8 °C, dilution 1:20, clone M-38c was obtained from the Developmental Studies Hybridoma Bank, created by the NICHD of the NIH and maintained at The University of Iowa, Department of Biology, Iowa City, IA 52242) and subseaquently with secondary anti-mouse antibody conjugated with AlexaFluor® 488 (45 min RT, dilution 1:300, A10667, Life Technologies). Then the cell nuclei were stained with propidium iodide. The cells were visualized under a confocal microscope (Zeiss LSM 5 DUO), λex = 488 nm, λem = 515–535 nm for osteocalcin or collagen and, λex = 560 nm, λem > 575 nm for propidium iodide, obj. 20, zoom 2×.

### Statistical evaluation of experiments with cells

Either One-way ANOVA and Student-Newman-Keuls Method or t-test were used for statistical evaluation of biological tests. The level of significance was set at 0.05.

### Scaffolds mechanical properties testing

**Fig. 3:**
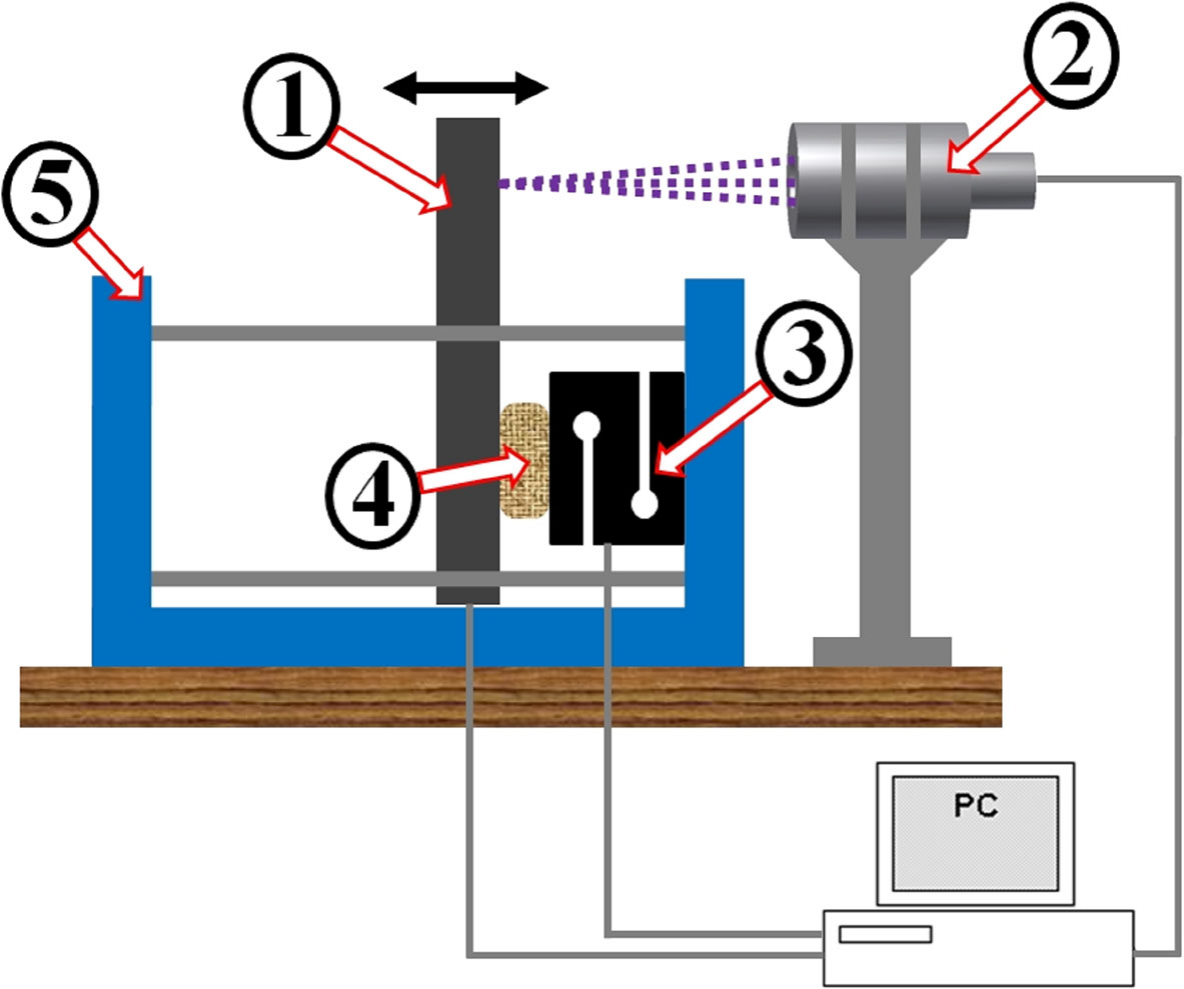
Apparatus served as a mechanism for scaffold’s loading. (1) Mobile cantilever driven by a stepper motor, (2) Confocal probe measures the cantilever displacement resp. scaffold deformation, (3) Force sensor, (4) Scaffold sample, (5) Stiff frame

As mentioned in scope of the research, scaffold with porosity 30% should provide better mechanical properties than scaffolds with higher porosity (50–90%). To validate whether this assumption is correct, it was necessary to perform the same mechanical testing for both of the scaffolds under the same conditions and then compare the results. The apparatus served as a mechanism for scaffold’s compressing and also for recording of force and displacement data. Individual parts which the apparatus consists of are described in Fig. 3.

### Devices and tools

Stepper motor used for cantilever movement, Long Travel Motorized Linear Stage 8MT295, Confocal probe Precitec CHRocodile M4, Force sensor RSCC – S-Type Load Cell – HBL.

### Measurement

Ten samples of each scaffold types (ST1, ST2) were used. The vertical thickness of each sample was measured before and after the deformation (after the load was applied and released – see Table 3). The load was applied by the cantilever directly on the scaffold sample, which was attached by oil adhesion to the force sensor – see scheme in Fig. 3.

A deformation of the scaffold is measured by a displacement of the cantilever immediately after it touches the scaffold sample. Force applied in time on the scaffold was measured by force sensor Force sensor RSCC – S-Type Load Cell – HBL A whole measurement process was recorded in time and transformed to a set of data that was then evaluated.

We have taken into account also a distortion of the measurement due to mechanical tolerance and compliance of the whole apparatus. The final measurement was performed without a scaffold sample and the displacement of the cantilever was measured as a function of force. This relation was then subtracted from the results measured when the scaffolds were used. As a result was obtained force-displacement relation of pure scaffold samples. The initial cross-section area of both types of scaffolds was similar. The accurate measurement of cross-section area of the scaffold was performed by ImageJ software.

Nominal instantaneous mechanical stress of samples was calculated as instantaneous force recorded by force sensor divided by initial cross-section area. Dimensionless deformation (engineering strain) of samples during loading was calculated as displacement divided by initial height of the sample.

To determine reasonable Young′s modulus, evaluated loading data range was 1.6–2 MPa which is close to stress of femur bone during normal gait as reported in discussion part related to this chapter. Moreover, in such a small range the deformation curve has almost linear behaviour, so the simple linear fit could be applied. Young′s modulus results are available in Table 4.

## Results

### Printing of scaffolds

The diameter of the scaffold fibres was set to 0.35 mm to meet the requirement for bone tissue regeneration. The geometry and inner structure of the scaffold ST1 were regular. Fibres exhibited flow in the gaps of the previous layer. Nevertheless, overall structure parameters enabled the scaffold to be used in cell seeding experiments.

**Fig. 4:**
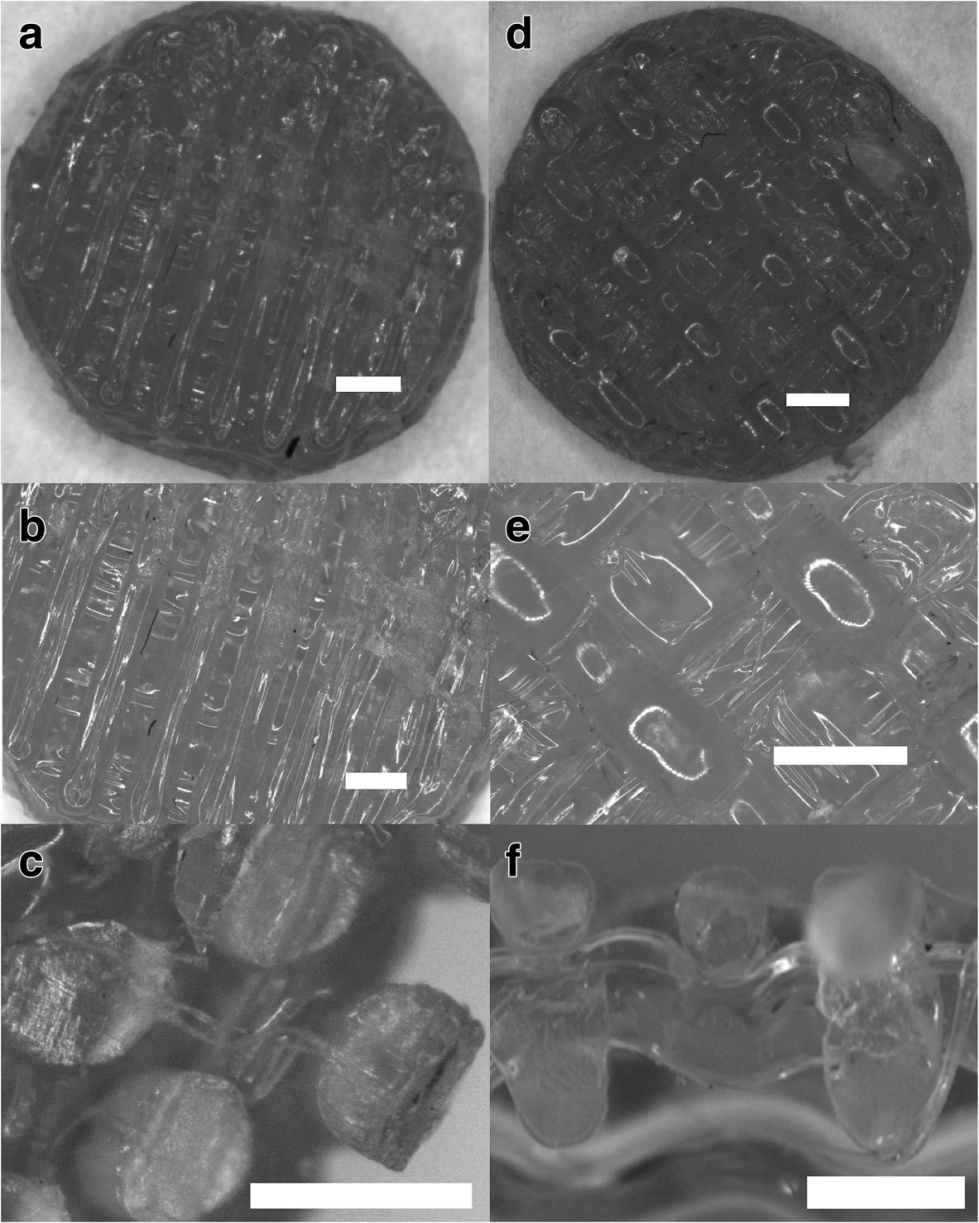
Structure description of printed ST1 and ST2. **a** Overall view of the scaffold ST1 from the top. **b** Detail of ST1 view from the top - Printed samples showed satisfactory external and internal geometry. **c** Sectional view of ST1 fibres. It can be seen that there is no porous or any other structural damages in an internal structure of the fibre. This is an important finding for the evaluation of mechanical properties of the overall scaffold. **d** Overall view of the scaffold ST2 from the top. **e** Detail of the view from the top - Printed samples showed satisfactory external and internal geometry. **f** Sectional view of the scaffold ST2. It can be seen that the precision of layering is of less quality than in the case of ST1 as the gaps between fibres are wider. Bar = 0.5 mm

For structure of ST2 the diameter of the fibre was set at 0.35 mm as in the case of ST1. The geometry and inner structure of the scaffold were regular. Fibres exhibited the same properties as in the case ST1 - a flow in the gaps of the previous layer. The overall structure parameters enabled the scaffold to be used in cell seeding experiments and for comparison of the results with ST1. Further comments on ST1 and ST2 scaffold structures are available in the descriptions of Fig. 4.

### Material characterization of scaffolds

The 3D printed scaffold was prepared from PLA.

Surface properties of PLA ware analysed using contact angle measurement and surface zeta potential. Contact angle of PLA was 74.3 ± 11.0° which corresponds to slightly hydrophilic surface. The wettability is essential for interaction with aqueous surfaces and for proper cell adhesion. In addition, the surface zeta potential plays important role for adhesion of proteins and formation of protein corona. Zeta potential on pure PLA surface was −40.6 ± 3.78 mV. The negative zeta potential is associated with binding of distinct proteins. In order to evaluate binding of proteins and molecules associated with bone regeneration, the PLA sample was incubated with type I collagen for 20 min. The analysis of surface zeta potential showed increase to −7.86 ± 2.64 mV. The change in surface zeta potential indicates that collagen binds to the surface of PLA samples. Similarly, the incubation with hydroxyapatite nanoparticles is associated with increase of zeta potential (−4.94 ± 1.54 mV) indicating the interaction with PLA surface. The both coated PLA had statistically higher zeta potential compared to uncoated PLA (*p* < 0.001). Determined molecular weight and polydispersity of used PLA were Mn (PLA) = 61,000 g/mol and Mw/Mn = 1.47, respectively.

### Scaffolds structure

The Table 1 below shows the calculated porosity of each individual scaffold, mean, median and SD of the set of values. T-test “Two-Sample Assuming Equal Variances” (alfa = 0.05) confirmed significant difference between ST1 and ST2.

**Table 1:** Determined porosity of both scaffold types

Scaffold no.	1	2	3	4	5	6	7	8	9	10	Mean	Median	SD
ST1	31%	32%	38%	27%	30%	33%	28%	35%	27%	34%	31%	31%	4%
ST2	52%	52%	46%	53%	44%	54%	54%	51%	53%	48%	51%	52%	3%

Three samples of each scaffold type were scanned by micro computed tomography (microCT) device which also allows for calculation porosity based on scanned 3D picture (see Table 2). The results correlate with results calculated from samples weight.

**Table 2:** Table presents the most relevant parameters gained from microCT

Parameter (dimension)	ST1a	ST1b	ST1c	ST2a	ST2b	ST2c
Total volume (mm^3^)	7.1	7.1	7.1	7.1	7.1	7.1
Solid volume (mm^3^)	5.1	2.4	1.8	3.3	7.6	8.7
All pores volume (mm^3^)	1.9	2.0	2.0	3.8	3.5	3.9
Closed pores volume (mm^3^)	0.0053	0.0063	0.0068	0.0002	0.0005	0.0001
Standard porosity (%)	27	28	28	53	50	55
Closed porosity (%)	0.075	0.090	0.096	0.003	0.008	0.001
Number of closed pores (1)	272	49	67	11	18	2
Surface of the sample (mm^2^)	49.3	43.8	44.5	37.3	38.8	38.7
Ratio of surface and volume (mm^−1^)	9.6	18.4	24.3	11.3	5.1	4.4
Average thickness of the fibres (mm)	0.36	0.41	0.39	0.36	0.36	0.36

**Fig. 5:**
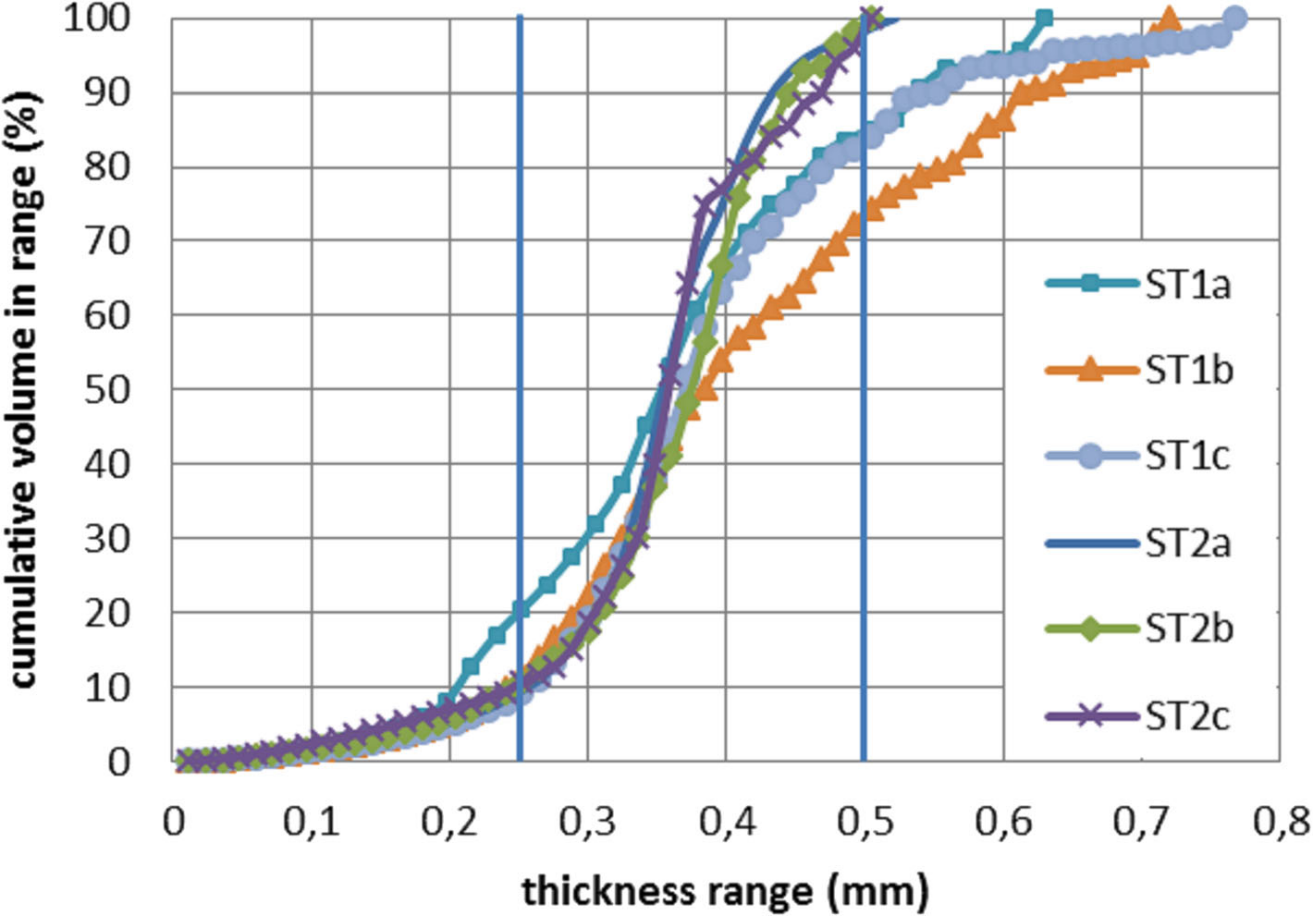
Fibre thickness distribution of ST1 and ST2 measured by micro CT. The thickness of fibres is not absolutely constant. Outlied values are likely residues of printing material (PLA), which is left on the sample when the printhead is moving from one side of the sample to another. A very thin fiber of PLA might be still leaking from the printhead during this movement

The average thickness of the fibres of both scaffolds was evaluated as 0.37 mm which corresponds to set of 3D printer. This assuming the pore size around 0.35 mm for ST1 and 0.7 mm for ST2. Nevertheless the thickness is not absolutely constant. The cumulative distribution of structure thickness corresponding to the volume which shows chart in Fig. 5.

**Fig. 6:**
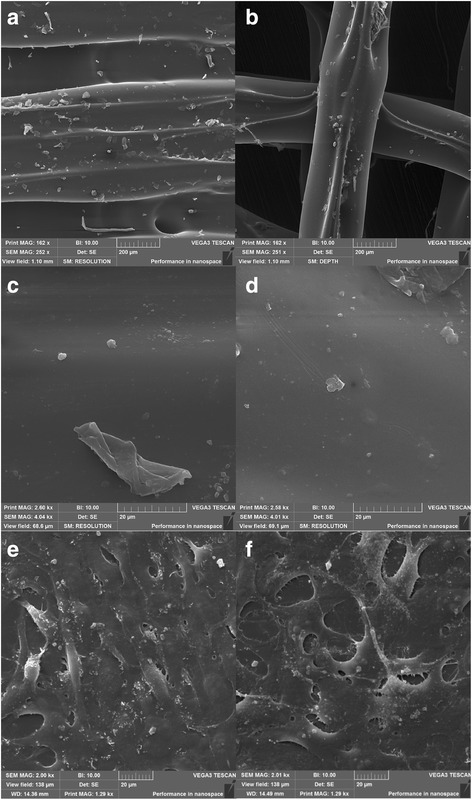
Scanning electron microscopy of ST1 (**a**, **c**, **e**) and ST2 (**b**, **d**, **f**) without and with cells. The surfaces of both scaffolds were smooth with irregular microparticles on the surface. Magnification × 250 (**a**, **b**), and × 4000 (**c**, **d**). Scanning electron microscopy of ST1 (**e**) and ST2 (**f**) seeded with osteosarcoma cells MG-63 after 2 days. MG-63 cells were spread on both scaffolds resembling oval to spindle-shaped morphology typical for osteosarcoma cells and forming small membrane protrusions. Magnification × 2000

In addition, topology of surface was analysed by SEM. Higher magnification of samples shows that the surface of 3D printed microfibers is made of smooth surface with minimal roughness. However, the surface also contains irregular defects in form of microparticles as defects from 3D printing process (Fig. 6, c and d).

**Fig. 7:**
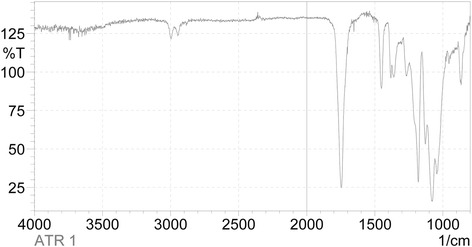
FTIR-IR spectrum of PLA. The CH3 group resonance was detected as peak at 2925 cm^−1^ and 1274 cm^−1^. The C = O group resonance was observed at 1756 cm^−1^, and carboxyl group was observed at 1090 cm^−1^

The chemical identity was analyzed using FTIR-ATR (see Fig. 7). The spectra showed samples typical for PLA. The CH3 group resonance was manifested as peak at 2925 cm−1 and 1274 cm−1. The C = O group resonance was observed at 1756 cm−1. In addition carboxyl group was detected at 1090 cm−1. The filament was made of PLA and does not contained significant contaminants.

### Experiments with osteosarcoma cells

The cell cytotoxicity test did not show significant differences between PLA conditioned medium and standard culture medium used for cell culture experiments. Therefore, PLA scaffold was considered not cytotoxic and was subsequently used for other cell culture testing.

**Fig. 8:**
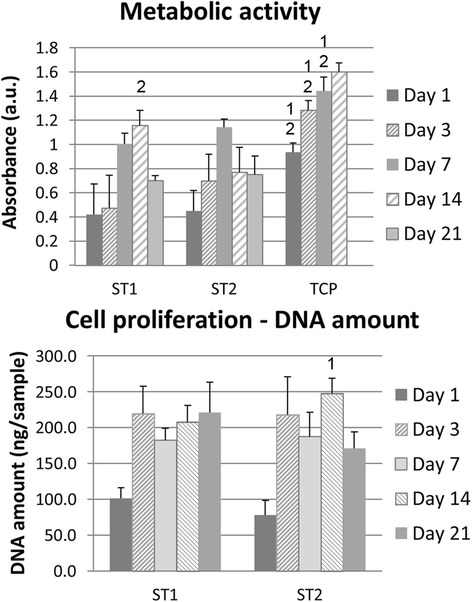
Metabolic activity and dsDNA. Metabolic activity and dsDNA amount are presented as mean of absorbance and standard deviation. Statistical differences compared to ST1 (1) or ST2 (2) groups are shown in graphs above SD values. Metabolic activity was higher on tissue culture polystyrene (TCP) compared to both scaffolds during 14 days; similar results were found for ST1 and ST2 scaffolds, except for higher absorbance on ST1 scaffolds compared to ST2 on day 14. Contrary, higher dsDNA amount was found on ST2 scaffolds than on ST1 scaffold on day 14

**Fig. 9:**
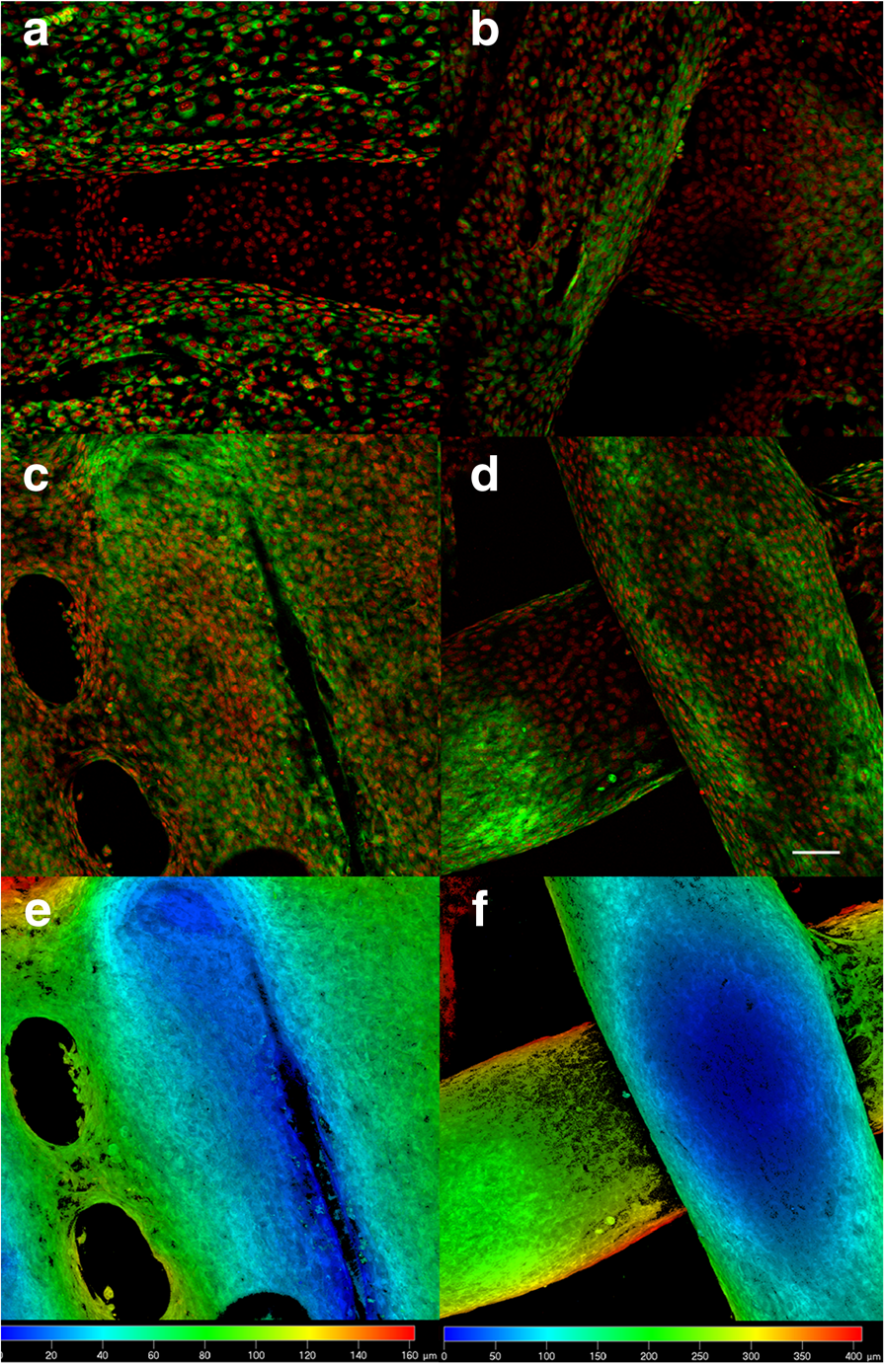
Confocal microscopy of MG-63 cells seeded on ST1 and ST2 - day 3 and day 7. Confocal microscopy of MG-63 cells seeded on ST1 (**a**, **c**, **e**) or ST2 (**b**, **d**, **f**) scaffolds from polylactic acid after a 3-day culture (**a**, **b**) or a 7-day culture (**c**-**f**). Cells were fixed and cell membranes were stained using DiOC6 (3) (*green*), cell nuclei were stained with propidium iodide (*red*). Both maximum projections (**a**-**d**) and color coded projections (**e**, **f**), which display depth (**d**) distribution of cells (d = 100 μm in E, d = 400 μm in F) showed fast growth of MG-63 cells on both scaffolds and formation of bridges from cells connecting fibres on ST1 scaffolds on day 7. Objective ×10, Magn. ×2, Bar = 100 μm

**Fig. 10:**
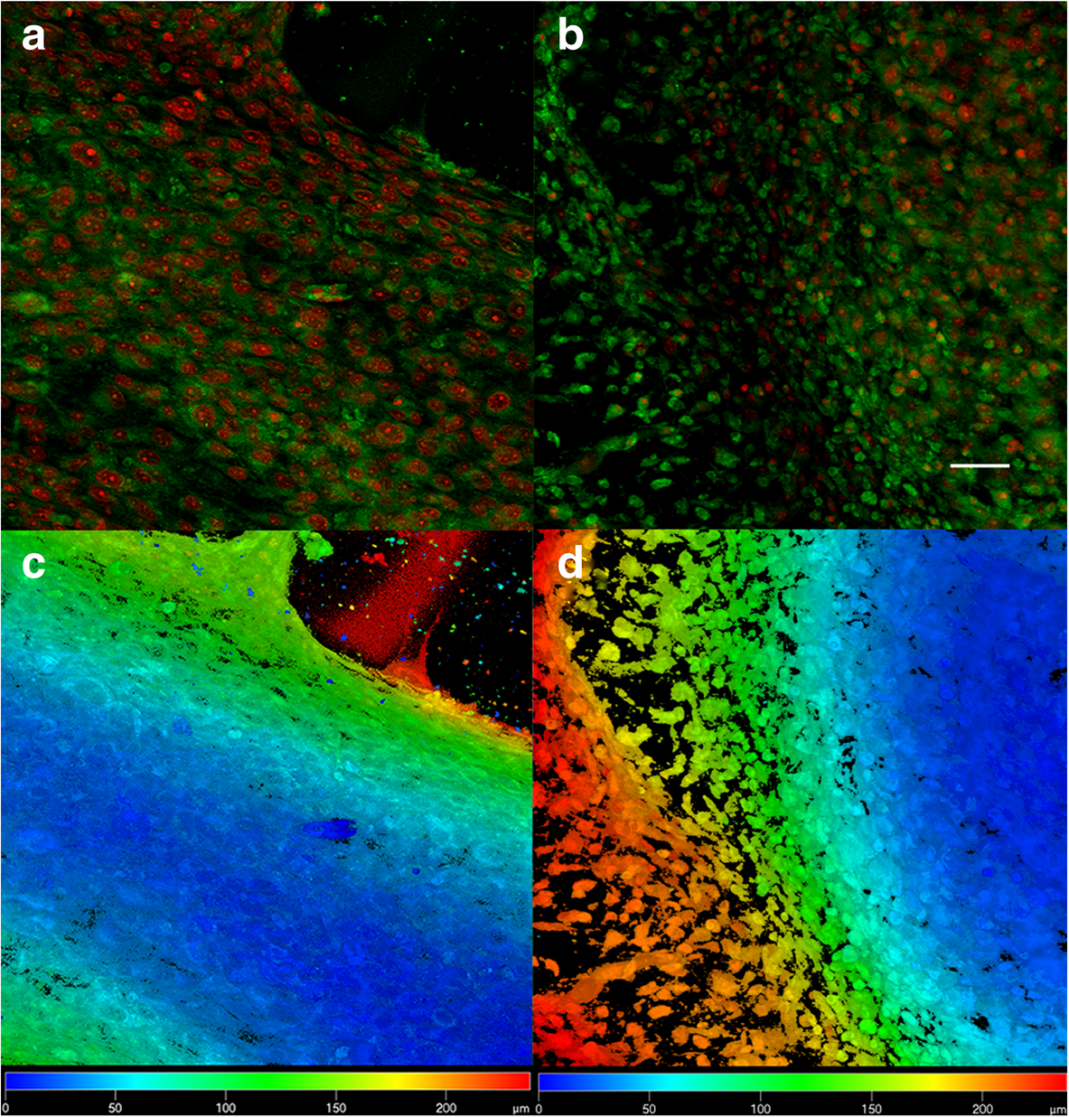
Confocal microscopy of MG-63 cells seeded on ST1 and ST2 - day 14. Confocal microscopy of MG-63 cells seeded on ST1 (**a**, **c**) or ST2 (**b**, **d**) scaffolds from polylactic acid after a 14-day culture. Cells were fixed and cell membranes were stained using DiOC6 (3) (*green*), cell nuclei were stained with propidium iodide (*red*). Both maximum projections (**a**-**b**) and color coded projections (**c**, **d**), which display depth (**d**) distribution of cells (d = 180 μm in C, d = 200 μm in D) showed confluent layer of MG-63 cells and formation of bridges from cells connecting fibres on both scaffolds. Objective ×10, magnification ×2, Bar = 50 μm

**Fig. 11:**
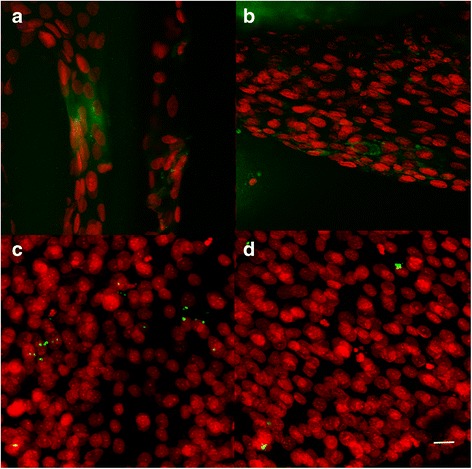
Confocal microscopy photomicrographs of ST1 and ST2 seeded with osteosarcoma cells. Confocal microscopy photomicrographs of ST1 (**a**, **c**) and ST2 (**b**, **d**) scaffolds from polylactic acid seeded with osteosarcoma cells MG-63 after a 7-day and 14-day culture. Immunohistochemical staining using monoclonal antibody against either type I collagen (**a**, **b**) or osteocalcin (**c**, **d**), followed by secondary antibody conjugated with Alexa Fluor 488® (*green*) and propidium iodide staining of cell nuclei (*red*) showed groups of cells producing type I collagen on both scaffolds (**a**, **b**) after 7 days, but only rare osteocalcin staining in both scaffolds (**c**, **d**) after 14 days. Objective ×10×, magnification ×4, bar = 20 μm

**Fig. 12:**
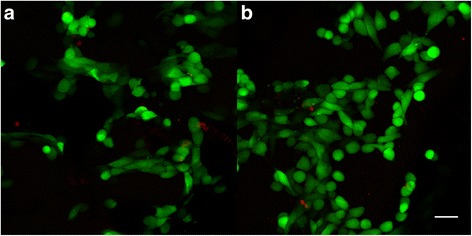
Live/dead staining of osteosarcoma cells seeded on ST1 and ST2 scaffolds. Confocal microscopy photomicrographs of live/dead staining of osteosarcoma cells seeded on ST1 and ST2 scaffolds after a 4-day culture. Live/dead staining of MG-63 seeded scaffolds showed high cell viability on both ST1(**a**) and ST2(**b**) scaffolds. Live cells (*green*), dead cells (*red*), objective ×10, magnification ×2, bar = 50 μm

The metabolic activity was highest on TCP, which is adjusted to optimum cell growth. ST1 scaffolds showed higher absorbance than ST2 scaffolds 14 days after seeding (Fig. 8). Fast cell growth was observed on both scaffolds on day 3 (Fig. 9a, b). This observation was in agreement with SEM method as on day 2 cells were confluently spread on the scaffolds surface (Fig. 6e, f). On day 7, there are visible cells “bridging” the gaps between individual fibres on ST1 scaffolds. Contrary, on ST2 scaffolds, cells are rather grouped around the cross joints of individual fibres. No bridging of gaps has apparently started yet. However, fibres are confluently covered by cells and the gaps between fibres are filled by cells on both scaffolds on day 14 (Fig. 10). Type I collagen is an early marker of bone differentiation. The staining after a 7-day culture showed type I collagen produced by cells on both scaffold. On the other hand, MG-63 cells produced only traces of osteocalcin, late marker of differentiation, on day 14 (Fig. 11). High cell viability was found on both scaffolds (Fig. 12).

### Results of mechanical tests

Following Tables 3 and 4 provide results of mechanical testing. Table 3 present vertical deformation testing results where non reversible deformations of all ST1/ST2 samples were compared using t-test “Two-Sample Assuming Equal Variances” (alfa = 0.05), and the result says that there is a significant difference between ST1 and ST2 as for the deformation properties. Table 4 shows calculated Young′s modulus of both scaffold types. Again, tested were 10 samples for each scaffold type and according to t-test “Two-Sample Assuming Equal Variances” (alfa = 0.05), there is a significant difference between ST1 and ST2.

**Table 3:** Vertical deformation of both scaffold types

Scaffold no.	1	2	3	4	5	6	7	8	9	10	Mean	Median	SD
ST1													
Height before vertical load h1 (mm)	1.34	1.45	1.54	1.28	1.31	1.47	1.43	1.54	1.20	1.45	1.40	1.44	0.11
Height after vertical load h2 (mm)	1.14	1.31	1.19	1.21	1.21	1.24	1.35	1.33	1.08	1.32	1.24	1.23	0.08
Non-reversible deformation Δ = (h2-h1)/h1 (%)	14.93	9.66	22.73	5.47	7.63	15.65	5.59	13.64	10.00	8.97	11.43	9.83	5.08
ST2													
Height before vertical load h1 (mm)	1.22	1.25	1.10	1.24	1.16	1.27	1.13	1.10	1.30	1.16	1.19	1.19	0.07
Height after vertical load h2 (mm)	0.94	0.87	0.94	0.92	0.99	0.81	0.78	0.75	0.94	0.90	0.88	0.91	0.08
Non-reversible deformation Δ = (h2-h1)/h1 (%)	22.95	30.40	14.55	25.81	14.66	36.22	30.97	31.82	27.69	22.41	25.75	26.75	6.83

**Table 4:** Young’s modulus of both scaffold types

Scaffold no.	ST1 (MPa)	ST2 (MPa)
1	56.8	13.54
2	67.8	22.06
3	27.9	47
4	43.22	16.14
5	51.9	59.9
6	34.2	23.4
7	51.17	20.33
8	38.7	27.8
9	32.6	20.02
10	51.9	19.41
Mean	45.619	26.96
Median	47.195	21.195
SD	11.80765	14.03683

## Discussion

Scaffold in tissue regeneration should be biocompatible and its properties should be tailored according to the tissue they regenerate. PLA is a biocompatible material used alone or as copolymers with other polymers, e.g. polyglycolic acid, poly-Ɛ-caprolactone, mainly for bone regeneration. The physical properties can be tailored by different methods of scaffold preparation, or using composite scaffolds. Besides this, modification with inorganic compounds or proteins follow in order to tailor physico-chemico properties and to improve cell growth or differentiation [4, 17, 21, 22].

Bio-fabrication techniques allow achieving fast, precise and cheap automatic manufacturing of 3D scaffolds. Rapid prototyping is a promising technique due to its high level of precision and controlling.

Based on the presented results of each particular experiment it is clear that the suggested approaches have demonstrated the ability to print biological scaffolds using the technologies in question. Furthermore, it was shown that designed PLA scaffolds allow proliferation and differentiation of cells, in this case osteosarcoma cells.

### Discussion related to printing of scaffolds

The reason for the oscillation of the fiber diameter along its length is apparently as follows - at the point of touch with the bottom fibre, the upper fibre is slightly flattened and the diameter (from the top view) is wider. In contrast, at the point of flow between the gaps of the bottom layer, the fibre is extended and the diameter is slightly reduced.

However, in terms of regularity, precision and porosity, the structure of both ST1 and ST2 scaffold is appropriate enough for cell proliferation.

### Material discussion

Many chemical parameters, e.g. chemical composition, charge, surface free energy or wettability are important for protein adsorption on the surface [17]. The adsorption of proteins present in culture medium, or blood is important for cell growth and differentiation. Highly hydrophilic materials did not support protein adsorption on the material surfaces; therefore they did not support cell adhesion which is mediated by adsorbed proteins from the medium or blood. On the other hand, proteins adsorb on highly hydrophobic surfaces in a rigid, denatured state, in which they do not support cell adhesion [23]. The evaluation of surface properties showed that our PLA 3D printed scaffolds were slightly hydrophilic. The result is in accordance with published literature [24]. Oppositely, Kao et al. [25], measured highly hydrophobic contact angle of PLA scaffold - 131.2° which was decreased to 51.9° by surface coating by poly (dopamine). Similarly, the addition of polyethylene glycol (PEG) or bioactive glass decreased the contact angle in PLA scaffold [21, 26]. However, the cell adhesion was showed to be optimal in samples with higher wettability. Khang et al. [27] showed that fibroblasts optimally adhered to modified PLGA sample with water contact angle of 53–55°. Similar results were observed in other studies [28, 29].

Zeta potential characterizes hydrophillicity of hydrophobicity of the material, and is influenced by chemical composition, charge, and morphology of the material [22]. The analysis by surface zeta potential showed highly negative values (−40 mV) of pure PLA surface. However, the cell adhesion is controlled by protein interaction with material surface. Bone extracellular matrix is predominantly composed of collagen I and hydroxyapatite. Collagen I and hydroxyapatite binding was analysed using surface zeta potential change. The surface zeta potential was in both cases significantly altered indicating binding to the surface of PLA. The results Hu et al. [30] showed that collagen is adsorbing to the surface of PLA film. The adsorbed collagen fibres are forming fibrous mesh on the surface of PLA. This may be important for optimal cell adhesion. The fibrous scaffold showed minimal surface roughness of fibres. Adsorption of collagen may improve adhesion of cells both in vitro and in vivo [22]. Similarly, hydroxyapatite is a key mineral component of bone tissue. In bone, type I collagen and other proteins or proteoglycans, e.g. osteocalcin, osteopontin, osteonectin, bone sialoprotein etc., are associated with inorganic components of bone, e.g. hydroxyapatite, calcium phosphates [21]. Zhang et al. [31] found that hydroxyapatite interacts with PLA with higher binding energy than with polymers without hydrophilic groups (carbonyl and carboxyl groups). Therefore, the surface properties of PLA scaffolds have potential for optimal osteoinduction. These properties combined with biodegradation in time-span of bone regeneration and customizable shape of implant predestinate the use of scaffold in bone tissue engineering.

### Discussion related to scaffold structure

Osteosarcoma cell line MG63 is often used to prove biocompatibility of the scaffolds as well as to test different microstructure or modifications of the scaffolds in vitro [32, 33]. They are usually used firstly in in vitro tests as they proliferate and express extracellular proteins in a standard way. On the other hand, mesenchymal stem cells (MSCs) show higher plasticity, their growth and ability to differentiate vary according to the cell origin and they provide more complex model in vitro tests. MSCs are often used for scaffolds of different composition or surface modification which are expected to alter both cell growth and mainly differentiation. The aim of the study was to test different methodology of PLA preparation and different architecture of the scaffold, which may have the biggest impact on the cell growth, diffusion of nutrition and cell viability.

Cell proliferation and differentiation are also affected by nanotopography, pore size, porosity, curvature of pores, and the rate of degradation [23, 32, 34, 35]. Pore size is an important parameter in 3D scaffolds. Minimum pore size that support cell ingrowth is considered to be 100 μm, although similar bone ingrowth was observed even in 50, 75, 100, and 125-μm holes of titanium triangle plate after its implantation into non-load bearing part of distal rabbit femur [36]. The porous scaffolds from poly (L-lactide-co-glycolide) with the same porosity but higher pore diameter showed higher cell penetration and cell proliferation after 1 week under static conditions compared to scaffolds with lower pores [32]. However, 300-μm or bigger pores are recommended for better vascularization and bone formation. On the other hand, smaller pores support osteochondral differentiation due to low vessel formation [37].

Cavo and Scaglione [38] performed computational modelling in order to optimize geometric pattern of 3D PLA scaffolds for cell ingrowth, fluid flow kinetics through the scaffolds. They found that pores of the diameter 600 μm and 300 μm interpore distances with 90° oriented interconnected pores formed scaffolds with the porosity of 52% and maximum flow velocity was found 1.1 cm/s which were the best among other tested scaffolds, including no interconnection of pores and 45°orientated interconnection. Further in vitro experiments proved higher cell number of primary human meniscus cells on scaffolds with 600 μm pore size compared to 900 μm pore size on day 3 and 5 after seeding.

In our ST1 scaffolds, fibre distance was about 350 μm, while in ST2 scaffolds the fibre distance was about 700 μm, which allowed higher cell growth after 14 days compared to ST1.

Interpretation of scaffold porosity calculated from its weight while density is known may be misleading, if absolutely closed pores are present significantly. Under such conditions scaffold seems to be highly porous, but cells are not able to adhere to closed areas and these areas stay unused. Because of this uncertainty, control measurement with scaffold samples were performed using high-accurate method microCT. The results confirmed that the number and especially the volume of closed pores are negligible in comparison to the volume of standard open pores. So called close porosity varied between negligible values 10^−3^% (ST2c) and about 10^−1^% (ST1c). The absence of closed pores should be advantage in the case of chemical sterilisation of the scaffold (sterilization medium may wet all scaffold surface). Ratio of surface to volume S/V was calculated as from 10 mm^−1^ to 24 mm^−1^ for ST1 and from 5 mm^−1^ to 11 mm^−1^ for ST2. For better imagination an endless cylinder with the diameter 0.35 mm has the ratio S/V 11.43 mm^−1^. S/V ratios of the samples are reduced due to connections between fibres. Distribution of the thickness of the scaffold structure may be interpreted as the most of scaffold material is incorporated in fibres in the diameter from 0.25 mm to 0.50 mm. Thus the structure is quite uniform.

Pore geometry is another important parameter that influences osteogenic differentiation. Killian et al. [39] reported that geometric features consistent with microenvironment of the differentiated cells increase actomyosin contractility and thus promote osteogenesis.

Zeta potential is the potential measured on the boundary of stationary and diffuse layer. Therefore it reflects also partial charge on the material surface. PLA does not have free charge in terms of having dissociated bonds, but the surface groups are partially negatively charged generating a negative zeta potential. The results are consistent with measured values for PLA nanoparticles ie. in Fischer et al. 2014 [40] notably, for PLA nanoparticles prepared without a surfactant a zeta potential of −49 mV was reported.

Surface macro- micro- or nano-roughness plays also role in cell adhesion, growth and differentiation. Nano-roughness of the surface supports cell adhesion and growth. Micro-roughness (100 nm – 100 μm) was shown to improve osteogenic differentiation of the cells [22, 35]. Jo et al. [41] fabricated polycaprolactone/pluronic F127 (PCL/F127) scaffold using 3D bio-printing and compared it with polycaprolactone scaffold. The PCL scaffold exhibited no pores in its strands but the PCL/F127 scaffold included nano- (∼200 nm) and micropores. Although the PCL/F127 scaffold had a lower compressive strength than the PCL scaffold, the surface of the PCL/F127 scaffold was after experiment fully (better than PCL) covered by cells due to its enhanced surface properties. Surface modification of 3D polycaprolactone by O_2_ plasma treatment led not only to increased hydrophilicity as well as to increased micro/nanoroughness of the surface which further slightly decreased by polymerization of acrylic acid on plasma-treated surface and by collagen immobilization on the surface. All treated surfaces increased metabolic activity of osteoblastic cell line in a MTT test [42]. Oxygen plasma and also nano hydroxyapatite are apparently useful techniques to improve the cell affinity. Roh et al. [43] showed in their study that the nano HA and O_2_ plasma surface treatment for PCL/nano HA composite 3D scaffolds enhanced the cell seeding efficiency, proliferation, and differentiation of MC3T3-E1 cells.

In our scaffolds surface was covered by small portion of microparticles originated from the preparation process which positively influenced cell growth. Further surface modification by collagen, fibrin, laminin, fibronectin or other proteins may be applied on prepared 3D scaffold; proteins form nanostructure containing natural binding sites which improve cell adhesion [38, 22]. Moreover, the addition of inorganic materials improved its osteinductive properties of the scaffolds [21].

Huang et al. [34] prepared composite poly-L-lactic acid (PLLA) – nano hydroxyapatite (nanoHA) porous scaffolds using low temperature rapid prototyping method. The scaffolds structure resembled foams with high range of pore diameter in the scaffolds. Interestingly, the pure PLLA scaffolds possess similar porosity – 55% as our ST2 scaffold. However, the addition of nanoHA increased the porosity up to 85% in 20% nanoHA scaffolds and afterwards decreasing to 72% for 40% nanoHA PLLA. Similar course was observed for pore diameter with the maximum of 392 μm in 20% nanoHA PLLA. This may positively have influenced the increased proliferation of rat osteoblasts on scaffold with 20% nanoHA PLLA along with nanostructured HA. On the other hand, the increased concentration of nanoHA from 10 to 40% significantly decreased tensile strength of the composite scaffolds. The addition of porogen is useful to enhance pore size, however, Thanh et al. [44] reported significantly higher both degradation of scaffold and acidification of simulated body fluids solution in porous PLA scaffold enriched with 20% nanospherical hydroxyapatite (HA) doped with magnesium and zinc and porogen compared to the scaffolds without NH_4_HCO_3_ porogen. Moreover, the addition of porogen was accompanied with decreased Young’ modulus by 78% in samples with 50/50 scaffold/ porogen ratio. These results show that the porosity of the scaffold shold be tailored very carefully with regard to both biomechanical and biological properties of the scaffolds. Simulated body fluid was used for deposition of HA on the scaffolds [44, 45]. Park et al. reported positive effect of HA deposited on patterned polycaprolactone scaffold on osteogenic differentiation of adiposed-derived stem cells [45]. Similar positive effect on MSC osteogenic differentiation was observed on decellularized tissue treated with HA-supersaturated solution [46]. Promising approach how to stimulate ostegenesis and support any new tissue formation as such may be an adsorption of plasmid DNA complexes onto a scaffold [47].

Chou et al. [48] developed composite scaffold based on PLA 3D–printed cage filled with corticocancellous bone. His composite scaffolds led to lower number of breakage of anterior cortical bone accompanied with leg shortening and deformation and higher rabbit activity during first 1 week postoperatively compared to controlled defects filled with chips of corticocancellous bone. Moreover, no over-inflammatory reaction and good bone regeneration was observed in all rabbits after 24 weeks.

Thermoplastic polymer PLA can be also combined with hydrogels. Rogina et al. [49] prepared 3D PLA scaffold by a fused deposition modelling system using a 3D Touch Double Head printer. The diameter of the lamellae was 400 um and the pore size up to 1000 um and a porosity about 60%. The composite chitosan-hydroxyapatite-PLA scaffold was prepared by freeze gelation technique. The composite chitosan-hydroxyapatite scaffold showed the highest mechanical stiffness as well as human mesenchymal stem cells (hMSC) proliferation, the slowest degradation compared to PLA and chitosan-PLA scaffolds. Moreover, osteoblastic markers osteocalcin and bone sialoprotein showed significantly higher gene expression compared to PLA scaffolds.

Dong Nyoung Heo et al. [18] 2017 reported 3D printed PLA scaffold combined with gelatin hydrogel which was functionalized with bioactive gold nanoparticles conjugated with cyclic arginine-glycine-aspartate (RGD). Non cytotoxic effect of the nanoparticles was observed while addition of RGD stimulated cell viability, proliferation and osteogenic differentiation of human adipose-derived stem cells. The compressive modulus of PLA scaffolds with 1.2 mm fiber spacing modified with gel and gold nanoparticles was comparable with mandibular bone; however, gel present in PLA scaffold did not improved compressive modulus.

Composite scaffolds from both synthetic and natural polymers have been tested. The composite porous scaffold prepared by modification of poly (3-hydroxybutyrate-co-3-hydroxyvalerate) with chitin nanocrystals led into scaffold with improved stiffness and attachment of adiposed-tissue derived cells compared to unmodified scaffold [50].

PLA is biocompatible material that is already used in clinical praxis as bone filler [51, 52]. The metabolic activity assay is influenced by both the number of cells and metabolic activity of mitochondrial enzymes. Both absorbance in MTS test and DNA amount increased on the scaffolds during culture more almost three times and 2.5-times, respectively, compared to the day 1. The cells proliferated well on both PLA scaffolds, which proved good biocompatibility of PLA scaffolds as we expected. From day 7 areas with confluent cell layer on the PLA surface were observed on both scaffolds. According to images taken by SEM, MG-63 adhered and spread on both PLA scaffolds with no observed differences on day 2.

The decrease of metabolic activity on ST1 and ST2 scaffolds on day 14 or 7, respectively, was related to full occupation of free spaces and reaching of optimal confluence. In additon, in occupied scaffolds the cells had lower access to nutrients and oxygen resulting in their decreased metabolic activity under static culturing conditions. Moreover, we have observed some detached cells from confluent cell layer on TCP samples during medium exchange from day 10, while adjacent cells migrated into free space and proliferated quickly.

Type I collagen is an early marker of differentiation while osteocalcin, non-collagenous protein, which is present in bone or dentin, is a late marker of differentiation [53, 54]. We have proved type I collagen formation of day 7 in both scaffolds. However, the amount of osteocalcin was negative on both ST1 and ST2 scaffolds on day 14.

According to the results it can be said that there was a slight difference between both structures in terms of cells proliferation, e.g. more porous ST2 scaffold supported better proliferation compared to ST1. Hypotheses stated at the beginning of experiment were therefore confirmed.

### Mechanical testing of the scaffolds

The idea was to empirically analyse whether the scaffold with lower porosity has lower deformation under the same load as the scaffold with higher porosity. ST1 has approximately 2 times lower range of vertical deformation than ST2 under the same loading (Table 3). It confirms logical assumption that if there is more material within the scaffold structure, the deformation is lower than in the case of a scaffold with less material within its structure. Another intention was to determine mechanical properties of each scaffold type and compare it with different scaffold types created for bone tissue replacement by different approaches and from different materials. Various “more or less complicated” ways how to describe and how to interpret mechanical properties of scaffolds are currently in use. When material properties and scaffold geometry are well known, finite element method (FEM) is being used to determine macroscopic relation between applied load and deformation response of a scaffold or maximum values of stress in fibre connections [55, 56]. The constitutive behaviour of scaffold material may be nonlinear and even time-dependent, especially in the case of polymers. Here we talk about viscoelasticity and description of such material requires more parameters. The golden standard of communication between engineers and medical doctors is a simple approach; scaffold structure is considered as homogeneous bar and stress-strain relation of a scaffold in the range of reversible deformation is interpreted as Young’s elastic modulus (tensile or compression). This parameter is nonlinear and depends on stress or strain level. If only one figure, not a graph, is required, it makes sense to consider the level of stress or strain corresponding to condition of intended use of the scaffold. Scaffolds in this study are intended to be used for bone tissue replacement. If we simplify femur bone as a tube with internal and external diameter 16 mm and 32 mm [57] and adopt value of axial load from ISO standard [58] for knee testing, which roughly simulates normal gait, the homogenized peek stress within cortical bone vary around 2 MPa.

Determined values of Young’s modulus of ST1 scaffold was 45.619 MPa and that of ST2 scaffold was 29.96 MPa. Presented values correspond with reported values for similar scaffold structures created from PLA material using 3D printing. Tiziano Serra et al. [17], in their article described several scaffolds where the Young′s modulus of the structures varies from 28 MPa to 93 MPa depending on their architecture. In case of different approaches of PLA scaffold fabrication, 3D printing seems to be more advantageous compared to e.g. freez-drying method as the 80% porous scaffold created by this method had the compressive Young′s modulus only 1.80 MPa [59]. Mentioned Young′s modulus ranges of PLA scaffolds are much lower than elastic modulus of bones; For example, cortical bone has a reported Young′s modulus in the range 1–20 GPa and a strength range of 1–100 MPa [60], with the equivalent values for cancellous (trabecular) bone of Young′s modulus 0.1–1.0 GPa and strength 1–10 MPa [61]. Such levels of Young′s modulus are reached rather by ceramics scaffolds fabricated by stereolitography. Sabree et al. [62] used stereolitography to fabricate scaffold with porosity at around 42% and Young′s modulus 2.9 GPa. Appuhamillage et al. [63] have shown how to overcome possible lack of adhesion at the interfilamentous junctions, resulting in non-uniform mechanical strength and its loss within FDM printed scaffold by blending PLA with a synthetic polymer containing Diels-Alder functionality. 3D scaffolds prepared by rapid prototyping can be properly functionalized with iron-doped hydroxyapatite nanoparticles with increased elastic modulus 650 MPa compared to 590 MPa of unmodified polycaprolactone scaffold [64]. Moreover, polycaprolactone scaffold with iron-doped hydroxyapatite nanoparticles positively influences the adhesion and growth of magnetically labeled MSCs compared to pure scaffold. These effects were enhanced with magnetic loading. On the other hand, the stress-strain diagram of iron-doped hydroxyapatite nanoparticles/polycaprolactone scaffold showed two platau-like regions that were not seed in pure polycaprolactone scaffold, which may be due to difference ductility of both scaffolds.

In this study, however, tested and reported are initial mechanical properties of scaffolds before degradation process and its further material processing in biological environment. Following the material properties of PLA, there are open questions which need to be investigated further. One of the questions is whether the scaffold structure would be appropriate for actual clinical application in bone regeneration engineering. The problem might be e.g. the amount of PLA material in relation to the volume of the scaffold. PLA naturally dissolves to lactic acid which is naturally present in the body, but too much of it might lead to pain and also local inflammatory responses during recuperation period [65]. On the other hand, the amount of the material must be sufficient to sustain supporting mechanical properties before enough amount of new tissue (bone) is created. Choon Peng Teng et al. [66] have synthetized highly porous star-shaped POSS-polycaprolactone-polyurethane (POSS-PCL-PU) as scaffold biomaterial for tissue engineering. In vitro degradation if this material was monitored during 52 weeks and exhibited slow initial weight loss of <1% during the first 2 weeks, followed by rapid weight loss of about 18% in the following 28 weeks. The material has also demonstrated excellent biocompatibility and rapid cell proliferation. Together with mechanical integrity, the degradation rate of such material can be controlled to achieve a scaffold that degradates slowly during the initial period and rapidly at the later phase after the growth of cells and desired tisse formation. Similar approach might be used also in the case of PLA. The timing of in vitro cultivation and in vivo implementation should therefore be one of the important points to investigate. To answer all these questions completely, it is clear that it would be necessary to perform a further series of experiments including the implementation into a living animal tissue, scaffold degradation testing and measurement, physiologically-mechanical tests during the degradation, etc. Such experiments outreach the scope of reported research. The other possibility is to use the scaffold for tissue cultivation in vitro only and implement the tissue in vivo after its full formation and after the full degradation of scaffold material. In such case the mechanical properties in terms of in vivo natural loading would not be important.

## Conclusion

Experiments in Tissue engineering focused on bio-fabrication of scaffolds were performed. We reported experiments focused on practical issues of bio-fabrication of scaffolds for tissue engineering in order to show how to possibly solve current technological limitations and issues in relation to printing of scaffold for bone tissue regeneration. Rapid prototyping technique based on Fused deposition modelling technique was used for fabrication of newly designed scaffold structures. Two types of scaffolds of the defined shape and engineered inner structure which provides regular and sufficient porosity have been successfully printed by ordinary commercial 3D printer. The diameter of the fibre of about 0.35 mm was achieved by tuning of the printing parameters. Presented layer size/filament diameter is still not the standard in current 3D printing, especially when using an ordinary 3D printing devices. Scaffolds were then seeded by osteosarcoma cells and our observations and measurements were focused on the toxicity of commercially available PLA used and its influence on cells viability, the proliferation of the cells and finally their ability to differentiate and provide osteoconductivity. The proliferation was satisfying and surprisingly equal for both scaffold types, even if the porosity values of the samples were 30% and 50% respectively, which confirmed new finding that it is likely not necessary to keep the recommended porosity of the scaffold for bone tissue replacement at around 90%. This fact also eliminates mechanical properties issues reported in case of scaffolds with high porosity because scaffold provided sufficient proliferation of cells and at the same time has more material within its structure, which ensures its better mechanical durability. Moreover, our scaffold ST2 with pore size about 0.7 mm demonstrated that the size of an individual pore could be almost double the size of the recommended range of between 0.2–0.35 mm without any effect on the proliferation.

These results should provide new valuable knowledge for further research and development in the field of scaffold bio-fabrication focused on bone tissue regeneration.

## Abbreviations

ATR: Attenuated total reflactance
DNA: Deoxyribonucleic acid
FBS: Fetal bovine serum
FDM: Fused deposition modelling
FEM: Finite element method
HA: Hydroxyapatite
hMSC: Human mesenchymal stem cells
MSC: Mesenchymal stem cells
nanoHA: nano-hydroxyapatite
PBS: Phosphate-Buffered Saline
PCL: Polycaprolactone
PEG: Polyethylene glycol
PGA: Poly (glycolic acid)
PLA: Poly (lactic acid)
PLGA: Poly (lactic-co-glycolic acid)
PLLA: Poly-L-lactic acid
RGD: Arginine-glycine-aspartate
SD: Standard deviation
SEM: Scanning electron microscopy
TCP: Tissue culture polystyrene
THF: Tetrahydrofurane

## References

[1] Hutmacher DW, Sittinger M, Risbud MV (2004). Scaffold-based tissue engineering: rationale for computer-aided design and solid free-form fabrication systems. Trends Biotechnology.

[2] Chanjuan D, Yonggang LV. Application of collagen scaffold in tissue engineering: recent advances and new perspectives. Polymers. 2016. doi:10.3390/polym8020042. 10.3390/polym8020042 PMC6432532 30979136

[3] Ha TLB, Quan TM, Vu DN, Si DM. Naturally derived biomaterials: preparation and application. Regenerative Medicine and Tissue Engineering. 2013. doi:10.5772/55668.

[4] Guntillake PA, Adhikari R (2003). Biodegradable synthetic polymers for tissue engineering. Eur Cell Mater.

[5] Munirah S, Kim SH, Ruszymah BHI, Khang G (2008). The use of fibrin and poly (lactic-co-glycolic acid) hybrid scaffold for articular cartilage tissue engineering: an in vivo analysis. Eur Cell Mater.

[6] Yang S, Leong KF, Du Z, Chua CK (2001). The design of scaffolds for use in tissue engineering—part I: traditional factors. Tissue Eng.

[7] Subia B, Kundu J, Kundu SC. Biomaterial scaffold fabrication techniques for potential tissue engineering applications. Tissue Engineering. InTech. 2010. doi:10.5772/8581.

[8] Chia HN, Wu BM. Recent advances in 3D printing of biomaterials. J Biol Eng. 2015; doi:10.1186/s13036-015-0001-4. 10.1186/s13036-015-0001-4 PMC4392469 25866560

[9] Lee VC. Medical applications for 3D printing: current and projected uses. Pharmacy and Therapeutic. 2014:704–11. PMC4189697 25336867

[10] Osama AA, Saied MD. Fabrication of tissue engineering scaffolds using rapid prototyping techniques. Engineering Technology Int J Mechanical, Aerospace, Industrial, Mechatronic Manuf Engineering. 2011;5:11.

[11] Polo-Corrales L, Latorre-Esteves M, Ramirez-Vick JE. Scaffold Design for Bone Regeneration. J Nanosci Nanotechnol. 2014:15–56. 10.1166/jnn.2014.9127 PMC3997175 24730250

[12] Ramtani S. Mechanical modeling of cell/ECM and cell/cell interactions during the contraction of a fibroblast-populated collagen microsphere: theory and model simulation. J Biomech. 2004; 10.1016/j.jbiomech.2004.01.028 15388313

[13] Ma PX, Elisseeff J. Scaffolding in tissue engineering. Biomed Eng Online. 2006. doi:10.1186/1475-925×-5-529.

[14] Velasco MA, Narváez-Tovar CA, Garzón-Alvarado DA. Design, materials, and Mechanobiology of biodegradable scaffolds for bone tissue engineering. BioMed Res Inter. 2015. doi:10.1155/2015/729076. 10.1155/2015/729076 PMC4391163 25883972

[15] Öchsner A, da Silva LFM, Altenbach H. Characterization and development of biosystems and biomaterials. Springer-Verlag Berlin Heidelberg. 2012. doi:10.1007/978-3-642-31470-4.

[16] Whang K, Healy KE, Elenz DR (1999). Engineering bone regeneration with bioabsorbable scaffolds with novel microarchitecture. Tissue Eng.

[17] Serra T, Mateos-Timoneda MA, Planell JA, Navarro M (2013). 3D printed PLA-based scaffolds: a versatile tool in regenerative medicine. Organ.

[18] Heo DN, Castro NJ, Lee SJ, Noh H, Zhu W, Zhang LG (2017). Enhanced bone tissue regeneration using a 3D printed microstructure incorporated with a hybrid nano hydrogel. Nano.

[19] An J, Teoh JEM, Suntornnond R, Chua CK. Design and 3D printing of scaffolds and tissues. Engineering. 2015; doi:10.15302/J-ENG-2015061.

[20] Rampichová M, Buzgo M, Míčková A, Vocetková K, Sovková V, Lukášová V, Filová E, Rustichelli F, Amler E (2017). Platelet-functionalized three-dimensional poly-ε-caprolactone fibrous scaffold prepared using centrifugal spinning for delivery of growth factors. Int J Nanomedicine.

[21] Vagaská B, Bacáková L, Filová E, Balík K (2010). Osteogenic cells on bio-inspired materials for bone tissue engineering. Physiol Res.

[22] Bacakova L, Filova E, Parizek M, Ruml T, Svorcik V (2011). Modulation of cell adhesion, proliferation and differentiation on materials designed for body implants. Biotechnol Adv.

[23] Von Recum AF, Van Kooten TG. The influence of micro-topography on cellular response and the implications for silicone implants. J Biomater Sci Polym. 1995; 10.1163/156856295x00698 7654632

[24] Navarro M, Engel E, Planell JA, Amaral I, Barbosa M, Ginebra MP (2008). Surface characterization and cell response of a PLA/CaP glass biodegradable composite material. J Biomed Mater Res A.

[25] Kao CT, Lin CC, Chen YW, Yeh CH, Fang HY, Shie MY (2015). Poly (dopamine) coating of 3D printed poly (lactic acid) scaffolds for bone tissue engineering. Mater Sci Eng C Mater Biol Appl.

[26] Serra T, Ortiz-Hernandez M, Engel ME, Planell JA, Navarro M (2014). Relevance of PEG in PLA-based blends for tissue engineering 3D-printed scaffolds. Mater Sci Eng C.

[27] Khang G, Lee SJ, Lee JH, Kim YS, Lee HB. Interaction of fibroblast cells on poly (lactide-co-glycolide) surface with wettability chemogradient. Bio-Medical Materials & Engineering. 1999; 10572622

[28] Choee JH, Lee SJ, Lee YM, Rhee JM, Lee HB, Khang G (2004). Proliferation rate of fibroblast cells on polyethylene surfaces with wettability gradient. Journal of Applied Polymer Science.

[29] Lee SJ, Khang G, Lee YM, Lee HB (2003). The effect of surface wettability on induction and growth of neurites from the PC-12 cell on a polymer surface. J Colloid Inter Sci.

[30] Hu AZ, Shi JB, Rong ZM, Xue P, Gong FR, Cheng SJ. Acta Polymerica Sinica. Adsorption Behav Collagen Spin-coated pla Surface. 2010; doi:10.3724/sp.j.1105.2010.09404.

[31] Zhang HP, Lu X, Leng Y, Fang L, Qu S, Feng B, Weng J, Wang J (2009). Molecular dynamics simulations on the interaction between polymers and hydroxyapatite with and without coupling agents. Acta Biomater.

[32] Pamula E, Filová E, Bacáková L, Lisá V, Adamczyk D (2009). Resorbable polymeric scaffolds for bone tissue engineering: the influence of their microstructure on the growth of human osteoblast-like MG 63 cells. J Biomed Mater Res A.

[33] Filová E, Suchý T, Sucharda Z, Supová M, Zaloudková M, Balík K, Lisá V, Slouf M, Bačáková L (2014). Support for the initial attachment, growth and differentiation of MG-63 cells: a comparison between nano-size hydroxyapatite and micro-size hydroxyapatite in composites. Int J Nanomedicine.

[34] Huang J, Xiong J, Liu J, Zhu W, Chen J, Duan L, Zhang J, Wang D (2015). Evaluation of the novel three-dimensional porous poly (L-lactic acid)/nano-hydroxyapatite composite scaffold. Biomed Mater Eng.

[35] Lossdörfer S, Schwartz Z, Wang L, Lohmann CH, Turner JD, Wieland M, Cochran DL, Boyan BD (2004). Microrough implant surface topographies increase osteogenesis by reducing osteoclast formation and activity. J Biomed Mat Res A.

[36] Itälä AI, Ylänen HO, Ekholm C, Karlsson KH, Aro HT. Pore diameter of more than 100 mu m is not requisite for bone ingrowth in rabbits. J Biomed Mater Res. 2001:679–83. 10.1002/jbm.1069 11745521

[37] Karageorgiou V, Kaplan D. Porosity of 3D biomaterial scaffolds and osteogenesis. Biomaterials. 2005:5474–91. 10.1016/j.biomaterials.2005.02.002 15860204

[38] Cavo M, Scaglione S. Scaffold microstructure effects on functional and mechanical performance: integration of theoretical and experimental approaches for bone tissue engineering applications. Mater Sci Eng. 2016:872–9. 10.1016/j.msec.2016.07.041 27524090

[39] Kilian KA, Bugarija B, Lahn BT, Mrksich M (2010). Geometric cues for directing the differentiation of mesenchymal stem cells. Proc Natl Acad Sci U S A.

[40] Fischer B, Heffeter P, Kryeziu K, Gille L, Meier SM, Berger W, Kowol CR, Bernhard K. Keppler. Poly (lactic acid) nanoparticles of the lead anticancer ruthenium compound KP1019 and its surfactant-mediated activation Dalton trans. 2014; 43:1096-1104. 10.1039/c3dt52388h 24165902

[41] Jo HH, Lee SJ, Park JS, Lee JH, Kim WD, Kwon SK, Lee JH, Lim JY, Park SA (2016). Characterization and preparation of three-dimensional-printed biocompatible scaffolds with highly porous strands. J Nanosci Nanotechnol.

[42] Park YO, Myung SW, Kook MS, Jung SC, Kim BH (2016). Cell proliferation on macro/nano surface structure and collagen immobilization of 3D polycaprolactone scaffolds. J Nanosci Nanotechnol.

[43] Roh HS, Myung SW, Jung SC, Kim BH (2015). Fabrication of 3D scaffolds with nano-hydroxyapatite for improving the preosteoblast cell-biological performance. J Nanosci Nanotechnol.

[44] Thanh DTM, Trang PTT, Thom NT, Phuong NT, Nam PT, Trang NTT, Seo-Park J, Hoang T (2016). Effects of porogen on structure and properties of poly lactic acid/hydroxyapatite nanocomposites (PLA/HAp). J. Nanosci. Nanotechnol.

[45] Park H, Lim DJ, Lee SH, Park H (2016). Nanofibrous mineralized electrospun scaffold as a substrate for bone tissue regeneration. J Biomed Nanotechnol.

[46] Yang M, Zhou G, Castano-Izquierdo H, Zhu Y, Mao C (2015). Biomineralization of natural Collagenous Nanofibrous membranes and their potential use in bone tissue engineering. J Biomed Nanotechnol.

[47] Zhao X, Komatsu DE, Hadjiargyrou M (2016). Delivery of rhBMP-2 plasmid DNA complexes via a PLLA/collagen electrospun scaffold induces ectopic bone formation. J Biomed Nanotechnol.

[48] Chou YC, Lee D, Chang TM, Hsu YH, Yu YH, Liu SJ, Ueng SW. Development of a three-dimensional (3D) printed biodegradable cage to convert Morselized Corticocancellous bone chips into a structured cortical bone graft. Int J Mol Sci. 2016; doi:10.3390/ijms17040595. 10.3390/ijms17040595 PMC4849049 27104525

[49] Rogina A, Pribolsan L, Hanzek A, et al. Macroporous poly (lactic acid) construct supporting the osteoinductive porous chitosan-based hydrogel for bone tissue engineering. Polymer. 2016:172–81.

[50] Li HY, Li H, Wang BJ, Gu Q, Jiang ZQ, Wu XD (2014). Synthesis and properties of poly (3-hydroxybutyrate-co-3-hydroxyvalerate)/chitin nanocrystals composite scaffolds for tissue engineering. Chinese Che Lett.

[51] Stancari F, Zanni B, Bernardi F, Calandriello M, Salvatorelli G. Use of PLA-PGA (copolymerised polylactic/polyglycolic acids) as a bone filler: clinical experience and histologic study of a case. Quintessence. 2000;51(1):47–52.

[52] Serino G, Biancu S, Iezzi G, Piattelli A (2003). Ridge preservation following tooth extraction using a polylactide and polyglycolide sponge as space filler: a clinical and histological study in humans. Clin Oral Implants Res.

[53] Born AK, Rottmar M, Lischer S, Pleckova M, Bruinink A, Maniura-Weber K (2009). Correlation cell architecture with osteogenesis: first steps towards live single cell monitoring. Eur Cells Mater.

[54] Gong Z, Wezeman FH (2004). Inhibitory effect of alcohol on osteogenic differentiation in human bone marrow-derived mesenchymal stem cells. Alcohol Clin Exp Res.

[55] Baldino L, Naddeo F, Cardea S, Naddeo A, Reverchon E. FEM modeling of the reinforcement mechanism of Hydroxyapatite in PLLA scaffolds produced by supercritical drying, for tissue engineering applications. J Mech Behav Biomed Mater. 2015. doi:10.1016/j.jmbbm.2015.07.021. 10.1016/j.jmbbm.2015.07.021 26275485

[56] Entezari A, Fang J, Sue A, Zhang Z, Swain MV, Li Q. Yielding behaviors of polymeric scaffolds with implications to tissue engineering. Mater Lett. 2016:108–11.

[57] Oftadeh R, Karimi Z, Villa-Camacho J, Tanck E, Verdonschot N, Goebel R, Snyder BD, Hashemi HN, Vaziri A, Nazarian A. Curved beam computed tomography based structural rigidity analysis of bones with simulated Lytic defect: a comparative study with finite element analysis scientific reports. Nature Publishing Group. 2016. doi:10.1038/srep32397. 10.1038/srep32397 PMC5009360 27585495

[58] ISO 14243 - Implants for surgery wear of total knee-joint prostheses Part 1: Loading and displacement parameters for wear-testing machines with load control and corresponding environmental conditions for test. International Organization for Standardization. 2009. https://www.iso.org/standard/44262.html. Accessed 3 Aug 2017.

[59] Chen X, Gu N, Yang HL, Zhang W, Luo ZP. Regulation of basalt fibers on PLA scaffold biodegradation. Orthopaedic research society. 2011. http://www.ors.org/Transactions/57/1914.pdf. Accessed 3 Aug 2017.

[60] Carter DR, Spengler DM. Mechanical-properties and composition of cortical bone. Clin Orthop Relat Res. 1978;9:192–217. 361320

[61] Gibson LJ (1985). The mechanical behaviour of cancellous bone. J Biomech.

[62] Sabree I, Gough JE, Derby B. Mechanical properties of porous ceramic scaffolds: influence of internal dimensions. Ceram Int. 2015:8425–32.

[63] Appuhamillage GA, Reagan JC, Khorsandi S, Davidson JR, Voit W, Smaldone RA. 3D printed remendable polylactic acid blends with uniform mechanical strength enabled by a dynamic Diels–Alder reaction. Polym Chem. 2017; doi:10.1039/c7py00310b.

[64] De Santis R, Russo A, Gloria A, D’Amora U, Russo T, Panseri S, Sandri M, Tampieri A, Marcacci M, Dediu VA, Wilde CJ, Ambrosio L (2015). Towards the design of 3D fiber-deposited poly (ε-caprolactone)/lron-doped Hydroxyapatite Nanocomposite magnetic scaffolds for bone regeneration. J Biomed Nanotechnol.

[65] Rahul MR, Amol VJ, Douglas EH (2010). Poly (lactic acid) modifications. Prog Polym Sci.

[66] Teng CP, Mya KY, Win KY, Yeo CC, Low M, He C, Yong HM. Star-shaped polyhedral oligomeric silsesquioxane-polycaprolactone-polyurethane as biomaterials for tissue engineering application. NPG Asia Materials. 2014; doi:10.1038/am.2014.102.

